# Supplementary material for: Magnetic Separation of Oxoacid of Boron from Salt-Lake Brine by Synergistically Enhanced Boron Adsorbents of Glucose-Functionalized SiO2 and Graphene
Source: Int J Mol Sci. 2022 Sep 26;23(19):11356. doi: 10.3390/ijms231911356 (PMC9570336; doi:10.3390/ijms231911356)
Supplement: Supplementary file 1 [file ijms-23-11356-s001.zip › ijms-1918803-supplementary.pdf]

Supplementary Materials

Magnetic Separation of Oxoacid of Boron from Salt-Lake Brine by Synergistically Enhanced Boron Adsorbents of Glucose-Functionalized SiO_2 and Graphene

Qinglong Luo [1,2], Xueying Wang [3], Mingzhe Dong [1,2], Xueli Huang [3,*], Zhijian Wu [1,2] and Jun Li [1,2,*]

[1] Key Laboratory of Comprehensive and Highly Efficient Utilization of Salt Lake Resources, Qinghai Institute of Salt Lakes, Chinese Academy of Sciences, Xining 810008, China
[2] Key Laboratory of Salt Lake Resources Chemistry of Qinghai Province, Qinghai Institute of Salt Lakes, Chinese Academy of Sciences, Xining 810008, China
[3] College of Chemical Engineering, Xinjiang University, Urumqi 830046, China
* Correspondence: xuelih@163.com (X.H.); junli@isl.ac.cn (J.L.)

.

Table and Figure

Table S1. Adsorption isotherms and kinetic equations.

Table S2. Composition of East Taigener salt-lake brine.

Figure S1. The EDS of the Go-Fe_3O_4@SiO_2-Glu.

Table S1.

Adsorption isotherms and kinetic equations.

	Models		Parameters
1	Langmuir adsorption isotherm	K_L	Langmuir adsorption constant
	$Q_e = \frac{Q_m K_L C_e}{1 + K_L C_e}$	Q_m	Final sorption capacity, mg/g
		Q_e	Sorptive quantity at liquid phase concentration C_e, mg/g
2	Freundlich adsorption isotherm	Q_e	Sorptive quantity at liquid phase concentration C_e, mg/g
	$log\, Q_e = log\, K_F + \frac{1}{n} log\, C_e$	Q_m	Final sorption capacity, mg/g
		K_F	Equilibrium constant
		n	Constant
3	Pseudo-first-order rate equation	Q_e	Sorptive quantity at equilibrium, mg/g
	$ln(Q_e - Q_t) = ln\, Q_e - k_1 t$	Q_t	Sorptive quantity at time t, mg/g
		k_1	Pseudo-first-order rate, min^{-1}
4	Pseudo-second-order rate equation	k_2	Pseudo-second-order rate, g/(mg·min)
	$\frac{t}{Q_t} = \frac{1}{k_2 Q_e^2} + \frac{t}{Q_e}$		
8	Intra-particle diffusion model	k_{id}	intra-particle diffusion rate constant, (mg g^{-1} $min^{-0.5}$),
	$Q_t = k_{id}\, t^{0.5} + I$	I	the boundary layer

Table S2.

Composition of East Taigener salt-lake brine.

Composition (g/L)	Na^+	K^+	Ca^{2+}	Mg^{2+}	Li^+	Cl^-	SO_4^{2-}	B
East Taigener salt-lake brine	77.65	18.860	0.176	34.01	0.969	180.77	75.798	0.807

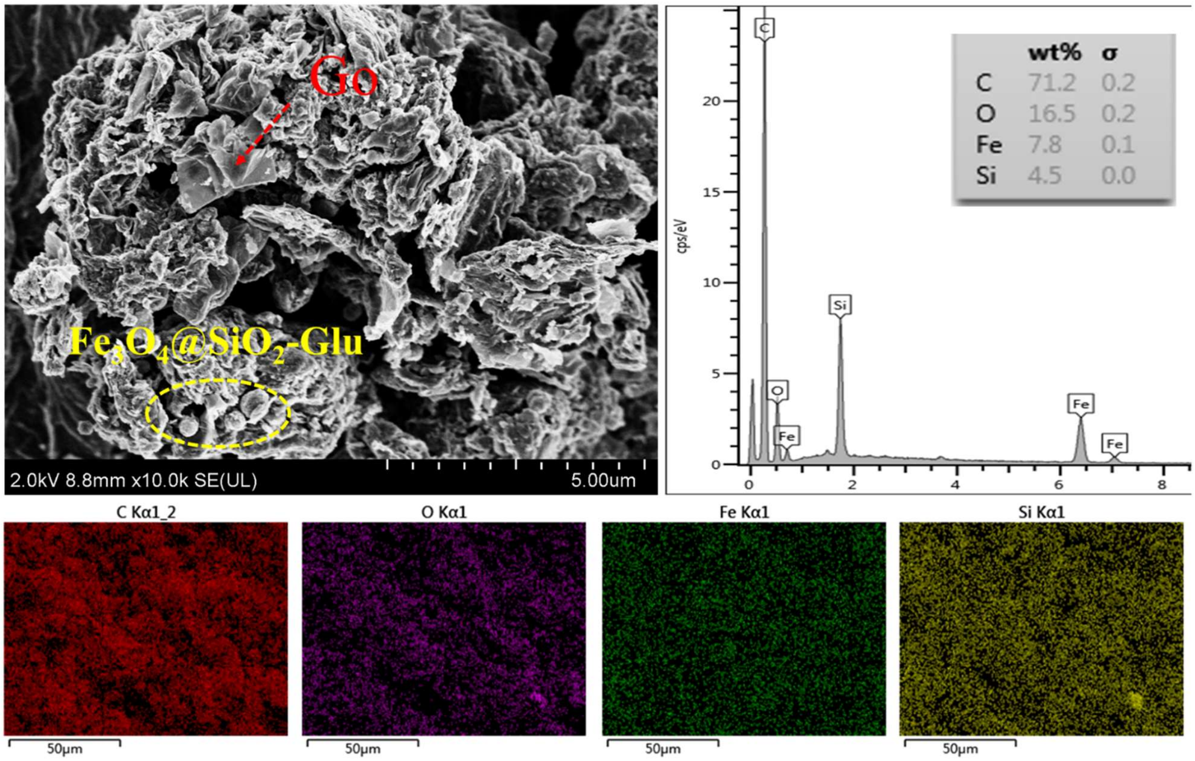

Figure S1. The EDS of the Go-Fe_3O_4@SiO_2-Glu.
